# The Social Determinants of Health, Health Disparities, and Health Justice

**DOI:** 10.1017/jme.2023.3

**Published:** 2022

**Authors:** Ruqaiijah Yearby

**Affiliations:** 1. THE OHIO STATE UNIVERSITY, COLUMBUS, OH, USA

**Keywords:** Health Justice, Employment, Health Disparities, The Social Determinants of Health, Home Health Care Workers

## Abstract

Although the federal government and several state governments have recognized that structural discrimination limits less privileged groups’ ability to be healthy, the measures adopted to eliminate health disparities do not address structural discrimination. Historical and modern-day structural discrimination in employment has limited racial and ethnic minority individuals’ economic conditions by segregating them to low wage jobs that lack benefits, which has been associated with health disparities. Health justice provides a community-driven approach to transform the government’s efforts to eliminate health disparities, by acknowledging the problem of structural discrimination; empowering less privileged groups to create and implement structural change; and providing support to redress harm.

## I. Background

For over twenty years, federal and state governments in the United States have tried to track and eliminate health disparities by addressing the social determinants of health (SDOH), which are social and economic conditions outside an individual’s control that limit an individual’s ability to reach their full health potential.^1^ In 2022, the federal government explicitly recognized that structural discrimination — macro-level conditions such as residential segregation — limits the conditions and well-being of less privileged groups, which keeps these groups from reaching their full health potential.^2^ However, federal and state governments have failed to adopt measures to eradicate structural discrimination in policies and plans aimed at addressing the SDOH and eliminating health disparities.^3^ Instead, they have adopted the health in all policies (HiAP) approach to integrate policy responses across sectors and used health impact assessments (HIA) to ensure decisions regarding laws and policies consider the health impacts.

Recent research has shown that the adoption of HiAP and use of HIAs has not resulted in broad changes in the SDOH or actual reductions in health disparities.^4^ Furthermore, neither the HiAP nor the HIA require the government to address structural discrimination, which research shows is one of the root causes of health disparities.^5^ For example, historical and modern-day structural discrimination in employment has limited racial and ethnic minority individuals’ economic conditions by segregating them to low wage jobs that lack benefits, such as paid sick leave and health insurance.^6^ This has been associated with health disparities.^7^ Health justice provides a community-driven approach to transform the government’s efforts to address the SDOH and eliminate health disparities.

The purpose of this commentary is to review my revised SDOH Framework, which includes structural discrimination as one of the root causes of health disparities, and discuss why governments should adopt the principle of health justice to address structural discrimination and eliminate health disparities.

Based in part on principles from the reproductive justice, environmental justice, food justice, and civil rights movements, health justice includes three guiding principles: 1) truth and reconciliation; 2) community-driven structural change; and 3) financial supports.^8^ By using these principles, the government can improve their efforts to address the SDOH and eliminate health disparities by acknowledging the problem of structural discrimination; empowering less privileged groups to create and implement structural change; and providing support to redress harm.

The purpose of this commentary is to review my revised SDOH Framework, which includes structural discrimination as one of the root causes of health disparities, and discuss why governments should adopt the principles of health justice to address structural discrimination and eliminate health disparities.^9^ This commentary proceeds as follows: Part II outlines my revised SDOH Framework, which includes many of the integral factors causing health disparities, such as structural discrimination and the law. Using home health care workers as a case study, Part III examines historical and modern-day examples of structural discrimination that have limited less privileged groups’ access to higher wages and benefits, which is associated with health disparities. Finally, Part IV discusses how the government (federal and state) can address structural discrimination and eliminate health disparities through truth, reconciliation, and community-driven structural change maintained with financial support.

## II. The Revised SDOH Framework

The current SDOH Framework includes key areas that limit an individual’s ability to achieve their full health potential, but it fails to show how structural discrimination is a root cause of differences in social and economic conditions between privileged and less privileged individuals. The work of public health and legal scholars — such as Mary Basset, Daniel Dawes, Gilbert Gee, Camara Jones, Nancy Krieger, Aysha Pamukcu, and Vernellia Randall — have shown “the totality of ways in which societies foster discrimination, via mutually reinforcing systems of discrimination (e.g. in housing, education, employment, earnings, benefits, credit, media, health care, criminal justice, etc.) that in turn reinforce discriminatory beliefs, values, and distribution of resources.”^10^

Structural discrimination — macro-level conditions such as residential segregation — limits the conditions and well-being of less privileged groups (including the disabled, poor, women, LGBTQIA+, the elderly, and racial and ethnic minorities), which keeps these groups from reaching their full health potential.^11^ Structural discrimination can be based on one attribute, such as gender identity, or multiple attributes such as race and class. Law (political process, statutes, regulations, policies, guidance, advisory opinions, cases, budgetary decisions, as well as the process of or failure to enforce the law) is one of the tools used to structure systems in a discriminatory way, which has been associated with health disparities. For example, structural discrimination in employment is evidenced by laws that allow employers to pay individuals with disabilities less than other workers, even if they are doing the same job.^12^ Structural discrimination limits the social and economic conditions of less privileged groups, which negatively impacts their health and well-being.

**Figure 1:**
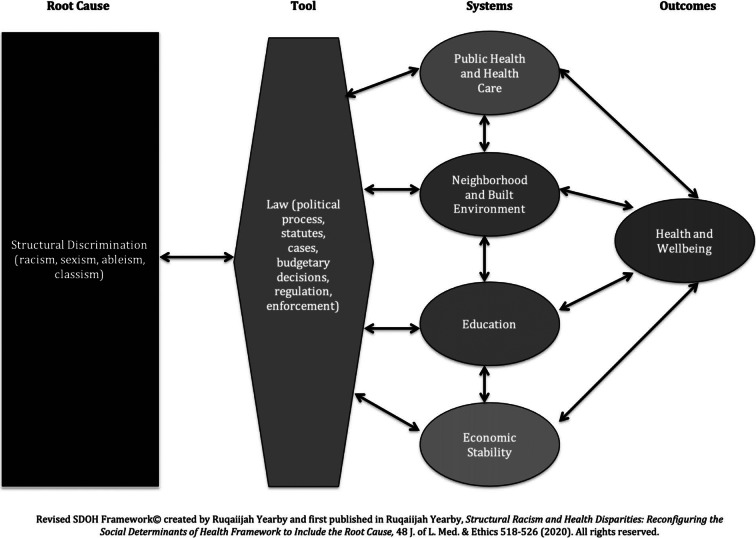

Numerous scholars have proposed methodologies and models to show how structural discrimination or racism is linked to health disparities. For example, Professors Chandra Ford and Collins Airhihenbuwa created the Public Health Critical Race Praxis methodology that noted structural determinism (which includes structural discrimination) and racial categories are the bases for ordering society, which contributes to health disparities.^13^ Additionally, David R. Williams et al. have created a model, entitled the house that racism built, showing how multiple forms of racism can affect health.^14^ Building on their work, my revised SDOH Framework, shown in Figure 1, highlights how structural discrimination is one of the root causes of health disparities.

My revised SDOH Framework provides a roadmap for how macro-level conditions are created that limit less privileged groups’ opportunities, resources, and access to healthy environments in four systems.^15^ Structural discrimination is a root cause of poor health outcomes for less privileged groups. Law is the tool used by the government to structure the four key systems (public health and healthcare, neighborhood and built environment, education, and economic stability) in a manner that disadvantages less privileged groups. These disadvantages limit the social and economic conditions as well as the health and wellbeing of less privileged groups compared to privileged groups. Using the example of home health care workers, who are predominately racial and ethnic minority women, this commentary demonstrates the connection between structural discrimination, law, systems, and health disparities.

## III. Employment and Health Disparities

Racial and ethnic minority women have been relegated to working in low wage jobs that lack benefits, such as paid sick leave and health insurance, in large part because of historical and modern-day structural discrimination. One example of this employment segregation is the high percentage of racial and ethnic minority women, who are employed as home health care workers (HHWs), which primarily includes personal care aides and home health aides.^16^ Home health care workers aid individuals with activities of daily living and perform clinical tasks such as taking blood-pressure readings, administering medication, and wound care.^17^ They are either employed by home health care agencies or directly by the patient to provide care in the patient’s home. Regardless of the employer, HHWs are classified as domestic workers. HHWs “make up a substantial share of the larger domestic worker job classification.”^18^ These workers remain in poverty, lack access to health care, and suffer significant health disparities as a result of laws that advantage business owners and White workers, while disadvantaging racial and ethnic minority women.

### A. Gender and Racial/Ethnic Makeup of HHWs

Laws passed after slavery were initially responsible for employment segregation. Specifically, many southern states passed “Black Code” laws after the end of slavery prohibiting Black Americans from working in any occupation other than domestic or agricultural service.^19^ Daneyelle Solomon et al. note “if [Black Americans] broke these laws or abandoned their jobs after signing a labor contract, they could be arrested,” imprisoned, and forced back into unpaid servitude on White plantations because of the 13th Amendment, which allows for slavery as punishment for a crime.^20^ Additionally, several laws were passed that prevented Black Americans from migrating to northern states to work in other industries.^21^

Consequently, throughout the late eighteenth and mid-nineteenth century, women of color, especially Black women, were relegated to working in the fields, cleaning houses, and watching children, which enabled White men and women to earn income and build wealth.^22^ Professor Peggie Smith notes, “as White, working-class women increasingly found jobs in the expanding industrial sector, domestic service became synonymous with Black women.” This is because of laws that structured the employment system in a discriminatory way that prevented Black women from obtaining jobs outside of domestic service.^23^

Between 1930 and 1940, “the percentage of White women employed as domestics declined from 37 to 11 percent, while the percentage of employed Black women in domestic service remained roughly constant, at slightly more than 50 percent,” allowing White men and women to work outside the home in higher paying jobs.^24^ Between 1950 and 1980, the percentage of Black women working in domestic service declined to between 11 to 5 percent.^25^ During this time, those working as HHWs also declined as the government prioritized providing care to the elderly and disabled outside of the home.^26^

The need for HHWs grew^27^ after the Supreme Court’s opinion in *Olmstead v. L.C.* in 1999,^28^ which required States to provide community-based services for people with disabilities. By the late 1990s, HHWs were more likely to be immigrants and to have earnings from other work, compared to nursing home aides.^29^ HHWs were also more likely to be in poverty (22%) than nursing home aides (16%) and the average population (12-13%).^30^ Today, many women of color, including Black women and Latinas, work as HHWs.^31^ In fact, almost two-thirds of all HHWs are racial and ethnic minority women.^32^ In southern states where “Black Code” laws were once popular, Black women are still “overrepresented in the home health care workforce compared to the overall labor force,” with Black women making up 43% of HHWs, but only 11% of all workers in the South.^33^

### B. Historical and Modern-Day Structural Discrimination in Employment Laws

In addition to laws supporting occupational segregation of women of color, many of the federal laws enacted during the 1930s to improve workers’ pay and benefits did not apply to domestic workers. For example, the Fair Labor Standards Act of 1938 (FLSA) limits the workweek to 40 hours and establishes federal minimum wage and overtime requirements.^34^ It also requires employers to keep records of the payroll. Although the FLSA did not explicitly bar racial and ethnic minority individuals from receiving these protections and benefits, it did *explicitly* exempt domestic workers, including HHWs, from these protections.^35^ In 1974, the FLSA law was amended to cover domestic workers, but those providing companionship services were exempted from these protections.^36^ HHWs, who were primarily women of color at that time,^37^ were determined to provide companionship services. Consequently, they were exempted from federal minimum wage and overtime requirements regardless of whether they worked for an individual patient or a home health care agency.^38^

In 2015, eighty years after the passage of the FLSA, the Department of Labor (DOL) issued regulations that for the first time made the FLSA apply to most HHWs.^39^ However, many workers still remain unprotected because under the Trump Administration, the DOL issued guidance allowing for many HHWs to be labeled as independent contractors.^40^ This is significant because the FLSA does not apply to independent contractors. Thus, the FLSA still does not apply to many HHWs, which means they do not receive the federal minimum wage or overtime pay. The federal government has also failed to provide HHWs with protections for collective bargaining activities, such as joining unions or negotiating for higher wages.

The National Labor Relations Act of 1935 (NLRA) provided collective bargaining protections for workers, allowing them to join unions, which resulted in higher wages and benefits, such as paid sick leave and health insurance.^41^ Workers covered by the NLRA, who join unions, are protected from being fired or punished for collective bargaining activities, such as negotiating for raises or benefits. However, the NLRA does not apply to domestic workers, which includes HHWs.^42^ Left without protection from the FLSA and NLRA, many HHWs remain in poverty.

A 2014 Fortune story listed the home health care job as one of the worst paying jobs in the United States.^43^ Although the Medicaid program^44^ primarily funds HHWs, the wages of these workers are so low that 1 in 5 (20%) are living below the federal poverty line, compared to 7% of all U.S. workers, and more than half rely on some form of public assistance including food stamps and Medicaid.^45^ Furthermore, many still work longer hours than other professions, without full pay, which has not changed much since the 1920s.

For example, “by 1925, while domestics regularly worked seventy-two to eighty-four hours a week, the average workweek for manufacturing workers declined from fifty-nine to fifty-hours a week.”^46^ However, domestic workers earned less than manufacturing workers and “other wage-earning women who worked in textiles, restaurants, shops, and factories.”^47^ In a 2019 study based on interviews of HHWs, some noted that they were working seventy-two hours a week, but were only being paid for thirty-six hours.^48^ Other studies found that 14% of HHWs work more than forty hours a week^49^ and many HHWs work more than one job.^50^ According to a 2021 Economic Policy Institute report, HHWs are paid on average $13.81 an hour nationally, which is approximately half of what an average U.S. workers is paid ($27.31).^51^ Additionally, a 2022 Economic Policy Institute blog found that in the south, where a majority of HHWs are Black women or Latinas, the average pay is less than $12 an hour.^52^

HHWs also lack health insurance. In 2021, almost 17% of HHWs were without health insurance and another 43% relied on Medicaid, Medicare, or some other form of public coverage.^53^ Approximately 26% of HHWs have employer sponsored health insurance, compared to 52% of all U.S. workers.^54^ In fact, most HHWs are “unable to afford their share of the health insurance premiums or they are ineligible for coverage because they work part time or are classified as independent contractors by their home health care agency.”^55^

The failure to provide HHWs with higher wages, overtime pay and collective bargaining rights, which provided access to paid sick leave and health insurance, is due to structural discrimination based on race and gender identity. Specifically, the government used the law, particularly the FLSA and NLRA, to structure the employment system in a discriminatory way, which has disadvantaged racial and ethnic minority women working as HHWs. Exempting these workers from the FLSA and NLRA has benefited White workers by boosting their wages and benefits, which has resulted in many of these workers entering the middle class.^56^ However, racial and ethnic minority HHWs remain in poverty because they are paid less than minimum wage, do not receive overtime pay, and lack benefits, such as paid sick leave. Seventy-seven years later, when most HHWs were finally covered by the FLSA, companies began classifying them as independent contracts. This benefits the companies by lowering employment costs, while harming workers who are left with low pay and without overtime pay or health insurance coverage. Lack of access to health insurance has been associated with health disparities, particularly for racial and ethnic minority women working as HHWs.^57^

### C. Health Disparities of HHWs

Few studies track the health of HHWs, but those that do show that HHWs not only have one of the highest rates of workplace injuries,^58^ but they also have worse general, physical, and mental health compared to other low wage workers.^59^ Because HHWs work alone in homes that are rarely designed for the proper delivery of healthcare services, HHWs tend to have higher rates of musculoskeletal symptoms, back injuries, and back pain than workers providing care in nursing homes.^60^ HHWs lost almost three times more workdays than nursing home workers due to injury, and were more likely to suffer “a slip, trip, or fall in the past year compared with therapists and office workers.”^61^

Moreover, “1 out of 4 HHWs rated their general health as fair or poor, 1 in 7 reported poor physical health, and 1 in 5 reported poor mental health,” which was significantly higher than health care aides and health support workers not working in the home.^62^ Low household income, history of depression, and an inability to see a doctor because of cost was associated with HHWs’ poor health status.^63^ Compared to health care aides and health support workers, HHWs were more likely to be women of color, have lower household incomes, and lack health insurance.^64^

Furthermore, because HHWs lack paid sick leave and the money to purchase personal protective equipment, they must continue to go to work in close proximity to patients that are often ill with infectious disease, like COVID-19, which has been associated with increased risk for contracting COVID-19.^65^ These health disparities are in large part due to structural discrimination in employment that has limited HHWs wages as well as access to health insurance and paid sick leave. Health justice provides principles that can be used to guide the government’s efforts to address structural discrimination and eliminate health disparities, which has harmed racial and ethnic minority workers, especially HHWs.

**Figure 2:**
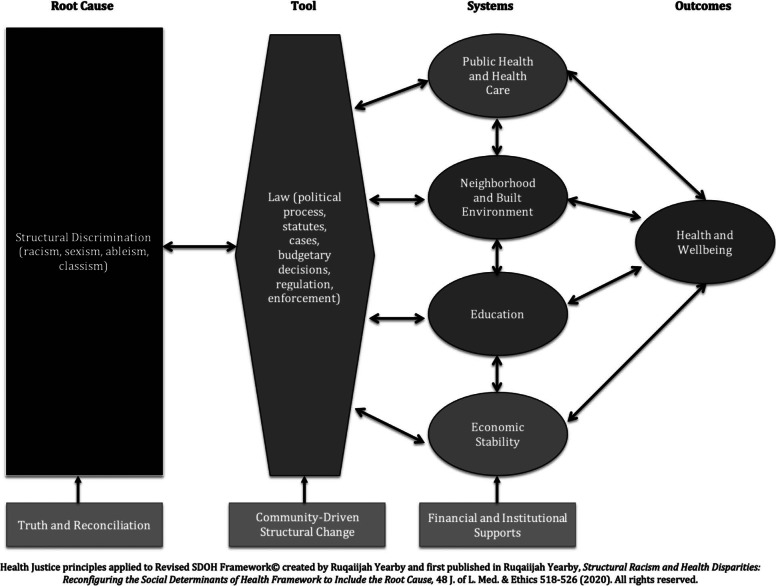

## IV. Health Justice

Unlike the current measures undertaken to address health disparities, such as HiAP and HIA, health justice provides a mechanism for systems-level change that will not only acknowledge the problem of structural discrimination, but also address the SDOH through community-driven action. Health justice is guided by three principles:^66^ (1) truth and reconciliation;^67^ (2) community-driven structural change; and (3) financial supports.^68^ As shown in Figure 2, each health justice principle provides a way to address the problems highlighted in my revised SDOH Framework.

*First,* to address structural discrimination, the root cause of health disparities, the government (federal and state) should implement a *truth and reconciliation* process that acknowledges the existence of structural discrimination and offers individuals from less privileged groups a mechanism to recover from the trauma of experiencing structural discrimination. Truth and reconciliation provide an opportunity for individuals from less privileged groups to heal and build trusting and respectful relationships with the government, which is necessary for meaningful community-driven change.^69^ Some governments have already adopted this process to address racial disparities.

For instance, Providence, Rhode Island adopted a *truth and reconciliation* process to address racial disparities, which began with the mayor and a group of advisers meeting to develop “a plan for sharing the state’s role throughout history in the institution of slavery, genocide of Indigenous people, forced assimilation and seizure of land; followed by city leaders reviewing laws and policies that resulted in discrimination against Black and Indigenous people; and concluded with community discussion about the state’s history and the ways in which historical injustices and systemic discrimination continue to affect society today.”^70^ This process should be used as a model. In the case of HHWs, the government needs to admit that its failure to apply the FLSA and NLRA to most HHWs and set Medicaid reimbursement rates at a level that provides a living wage for HHWs are examples of structural discrimination. The government (federal and state) then needs to provide HHWs with an opportunity to share their experiences and stories with the government, particularly policymakers and regulators.

*Second,* the government (federal and state) needs to change the laws (political process, statutes, regulations, policies, guidance, advisory opinions, cases, budgetary decisions, as well as the process of or failure to enforce the law) that structure systems in a discriminatory way. These changes must be done in partnership with individuals from less privileged groups to ensure that the laws address their needs. For example, some state governments have already formed an employment relationship with HHWs, providing the workers with the power to negotiate and obtain higher wages and benefits, such as health insurance.^71^ The state of Washington has been a leader in providing HHWs with the power to change the structurally discriminatory employment system.

After the Quality Home Care Initiative (Initiative 775) was passed by voters in 2001, HHWs were able to form a union named SEIU 775.^72^ As a result of the law, the State of Washington formed an employment relationship with HHWs and is required every two years to work with SEIU 775 to set the minimum wage rate for all HHWs, including those working for a home care agency and those working independently. In the first contract with the State, SEIU 775 was able to obtain health benefits for all HHWs. In the second contract, the State “included a wage scale with step increases for hours worked by home care aides, paid vacation time, workers’ compensation, and mileage reimbursement.”^73^ Nationally, some HHWs have even been able to develop Home Care Worker cooperatives, which are owned by HHWs. These cooperatives gives HHWs the power to negotiate better wages, benefits, and worker conditions.^74^ These examples should serve as a model for community-driven structural change where individuals from less privileged groups work in partnership with the government to transform systems.

*Third*, the government (federal and state) must provide financial supports to redress harm. Some governments have tried to provide financial support for individuals from less privileged groups using money from federal COVID-19 economic relief funds. The mayors of Mount Vernon, New York, and St. Paul, Minnesota, have used part of their economic relief funds to provide a guaranteed income program for some residents.^75^ This financial relief should be provided to all less privileged groups to offset the decades of unequal social and economic conditions they have experienced. For HHWs, this could be achieved by providing them with additional wages. In 2019, twenty-two states had adopted policies to provide direct care workers, which includes HHWs, with increased wages using wage pass through laws.^76^ Prior research has shown that these laws are a viable policy option for raising HHWs’ wages.^77^

Structural discrimination has resulted in centuries of unequal social and economic conditions for less privileged groups, which has limited their ability to reach their full health potential. This will take generations to fix. A 2016 research study found that “if the average wealth of a Black family continued to grow at the same pace it had over the past three decades, it would take Black families 228 years to amass the same amount of wealth White families had in 2016. That’s just 17 years shorter than the 245-year span of slavery in this country. For the average Latino family, it would take 84 years to amass the same amount of wealth White families had in 2013 — that’s the year 2097.”^78^ Thus, it is going to take a long time, even generations, of intentional work to eradicate structural discrimination and eliminate health disparities. During this time, governments must acknowledge the problem of structural discrimination; empower less privileged groups to create and implement structural change; and provide support to redress harm.

## Note

The author has no conflict of interest. The author wants to acknowledge the work of Danyelle Solomon, Connor Maxwell, Abril Castro, and Daniel Costa concerning historical structural racism and wage gaps for domestic workers, the work of Angela Harris, Aysha Pamukcu, Lindsay Wiley, and Emily Benfer defining health justice, as well as the work of PHI National and the Service Employees International Union to obtain rights for home health care workers.
